# Serum soluble interleukin-2 receptor (sIL-2R) is an accurate biomarker for dengue-associated hemophagocytic lymphohistiocytosis syndrome diagnosed by Hscore

**DOI:** 10.1007/s15010-022-01906-8

**Published:** 2022-08-23

**Authors:** Chakrapani Mahabala, Vivek K. Koushik, Poornima A. Manjrekar, Prashantha Balanthimogru

**Affiliations:** 1 Department of Medicine, Kasturba Medical College, Mangalore, 575001 India; 2 Department of Nephrology, Apollo Hospital, Chennai, India; 3 Department of Biochemistry, Kasturba Medical College, Mangalore, India; 4 Department of Medicine and Department of Adult Hematology, Kasturba Medical College, Mangalore, India; 5 Manipal Academy of Higher Education, Manipal, India

**Keywords:** Dengue fever, Hemophagocytic lymphohistiocytosis, HLH, sIL-2R, Ferritin, H Score

## Abstract

**Objective:**

Hemophagocytic lymphohistiocytosis is a potentially fatal complication of severe dengue fever. Here we evaluated the serum soluble IL-2R levels as potential biomarker for identifying HLH in patients with dengue fever.

**Methods:**

In this cross-sectional study conducted in a tertiary care center of a teaching hospital, subjects with dengue and fever of more than 5 days, leukopenia/thrombocytopenia and/or hepatosplenomegaly were studied. Data were collected to compare sIL-2R values and serum ferritin with Hscore and Histiocyte Society 2004 criteria. Relevant statistical methods were used.

**Results:**

80 subjects with severe dengue fever were analyzed with relevant investigations. Mean H score was 219.2 ± 17.6 in 18 dengue patients with HLH v/s 166.2 ± 11.2 in 62 patients without HLH (*p* =  < 0.001). Serum ferritin (11,230.5 v/s 7853.5, *p* = 0.013) and sIL-2R (32,917.5 v/s 6210, *p* = < 0.001) were significantly higher in those patients with HLH. sIL-2R correlated very well with HScore (*r* = 0.98, *p* < 0.001) compared to ferritin (*r* = 0.51, *p* < 0.001) with an AUROC of 1.00 compared to 0.694 (95% CI 0.557–0.831) of serum ferritin for diagnosing HLH. A cut-off value of 10,345 pg/ml for sIL-2R had a sensitivity and specificity of 100% for HLH, whereas, a ferritin value of 8613 ng/ml had only 67% sensitivity and 55% specificity.

**Conclusion:**

sIL-2R could be a single most useful biomarker to differentiate dengue fever patients who are likely to progress to HLH, from those that are not. Full workup for HLH could be limited only to those patients with elevated sIL-2R, especially in resource limited settings.

**Supplementary Information:**

The online version contains supplementary material available at 10.1007/s15010-022-01906-8.

## Introduction

Severe dengue is a major complication in dengue fever. Presence of hemophagocytic lymphohistiocytosis (HLH) in dengue fever is one of the inclusions for expanded dengue syndrome, which is a subset of severe dengue [1]. Dengue HLH is seen in about 17% of patients with severe dengue and has 2 times higher mortality compared to those without HLH (39 Vs 19.1%) [2, 3] hemophagocytic lymphohistiocytosis (HLH) is a hyper-inflammatory condition involving the innate immune system [4], which develops either as inherited inability to check the inflammation that is initiated by macrophages (Primary HLH) or because of certain infections, malignancies or rheumatologic conditions which stimulate the monocyte-macrophage system (Secondary HLH) [5]. HLH is underdiagnosed in dengue fever and early diagnosis and prompt monitoring and intervention might reduce mortality in dengue associated HLH [3].

Histiocyte Society criteria which were modified in 2004, are primarily for the diagnosis of childhood Primary HLH. They have not been validated for adult secondary HLH which includes infection-associated HLH. Subsequently, Fardet et al. developed a web-based score by assigning graded points for different parameters, and was developed and validated for primary as well as for secondary HLH [6, 7]. Performance of HScore is better than the Histiocyte Society criteria for secondary adult onset HLH [8]. Patients with dengue fever commonly develop leukopenia and thrombocytopenia, hepatomegaly and altered liver enzymes. Since these abnormalities are seen in HLH also, transition from severe dengue to HLH dengue is difficult to recognize. In the actual clinical settings, treatment of HLH should be initiated if there is strong suspicion of HLH without waiting for complex and expensive diagnostic procedures since delay in initiating the treatment will lead to irreversible organ damage [8, 9]. Availability of a single parameter which can diagnose HLH in patients with dengue fever could serve as a simple biomarker in management of these patients.

Interleukin 2 receptors are heterotrimeric transmembrane proteins [10], which are expressed on the cell surface of T lymphocytes when they are stimulated. Enzymatic cleavage of the alpha component leads to release of this component of the receptor into blood as soluble Interleukin 2 receptor (sIR-2R) which is also known as soluble CD 25(sCD25) [10, 11]. Hence, serum soluble IL-2R levels are surrogate for activated T cell activity which is the primary abnormality in HLH [12]. Rising levels of sIL-2R precede other clinical and hematological parameters of hyper-inflammation [13]. Previous studies have evaluated the utility of sIL-2R in diagnosing HLH, but were conducted in heterogeneous mixed disease etiologies and/or consisted of HLH cases diagnosed by Histiocyte Society 2004 criteria [10, 12]. Since sIL-2R is part of the diagnostic criteria in the Histiocyte society criteria, there can be confounding by insertion bias in these analyses. Defining HLH by HScore [14], which does not include sIL-2R, would be a suitable alternative. The diagnostic yield might be better if homogeneous disease entity is studied. Hence the present study was undertaken to evaluate the role of sIL-2R as potential biomarker for identifying HLH in patients with dengue fever diagnosed by HScore. Unlike Histiocyte Society criteria 2004, which gives binary outcome as presence or absence of HLH, HScore gives the probability of HLH in a given patient [6]. Initial reports by Fardet et al. suggested a cut-off score of 169 for the diagnosis of HLH. H score of more than 185 was chosen in our study based on the study done by Debaugnies F et al. In this retrospective study, cut-off of 185 yielded a sensitivity of 85% and Specificity of 88% for the diagnosis of HLH in adult patients based on the judgment of the treating team and confirmed by 2 independent experts who had more than 10 yrs of experience in managing patients with HLH [15]. 14/20 (70%) patients with HLH had hemophagocytosis in the marrow compared to 24/48 (50%) among patients without HLH. Bone marrow is neither sensitive nor specific for HLH and it may be absent in early stages of acute infection induced HLH where it might be apparent in subsequent examinations. HScore of 185 roughly translates to about 80–85% probability of HLH, whereas score of 169 gives only 65% of probability of HLH [6].

## Materials and methods

In this cross-sectional study conducted in a tertiary care center of a teaching hospital, subjects with dengue and fever of more than 5 days, leukopenia/thrombocytopenia and/or hepatosplenomegaly were studied. Data were collected to compare sIL-2R values with HScore and Histiocyte Society 2004 criteria. Analysis pertaining to HScore is presented here. Dengue fever was diagnosed by NS1 antigen and/or IgM antibody by ELISA. Patients underwent blood tests for deriving HScore. An additional blood sample was stored at – 80 °C for measuring sIL-2R. HScore was derived using the web-based HScore calculator [14] at http://saintantoine.aphp.fr/score/ as previously described by Fardet et al. [6]. Ten out 12 parameters of this online calculator were available for calculating the score. Bone marrow examination and fibrinogen levels were not available in this study. HLH was diagnosed if HScore was more than 185 [15]. The DIACLONE CD25/IL-2R ELISA kit which is a solid phase sandwich ELISA, was performed as per kit manufacturer’s instructions for estimating sIL-2R levels. The minimum detectable dose of IL-2R using DIACLONE CD25 ELISA kit was 32.5 pg/mL. Normal level of sIL-2R, according to the kit manufacturer, is 4051 (SD 1998) pg/mL. Nine samples had extremely high sIL-2R values (close to 46,990 pg/mL). Further dilution was not done in these samples. Hence, maximum values were expressed as ≥, in the tables. The study was approved by Institutional Ethics committee and Informed consent was obtained from all study participants.

Continuous variables were expressed as Median, IQ range, and Minimum–Maximum range since most of the parameters were not normally distributed, and categorical variables were expressed as frequency (%). Comparison of Median between patients with HScore < 185 and those with score > 185 was done using Mann–Whitney *U* test (nonparametric). Categorical parameters were compared using chi-squared test. *p* < 0.05 was considered as significant. Correlation coefficient was calculated by Spearman’s method to determine correlation of sIL-2R and serum ferritin with HScore. ROC curve analysis was performed to evaluate the efficacy of sIL-2R and serum ferritin to identify HScore of > 185. Sensitivity and specificity of sIL-2R and S. ferritin in diagnosis of HScore > 185 were calculated. sIL-2R levels were compared between those with severe dengue and non-severe dengue. sIL-2R levels between males and females were also compared by nonparametric methods. Correlation coefficient of sIL-2R with other common parameters like platelet count and hematocrit was also calculated. Comparison of performance of sIL-2R was done with serum ferritin since ferritin is a routinely performed test for diagnosing HLH.

## Results

**Fig. 1:**
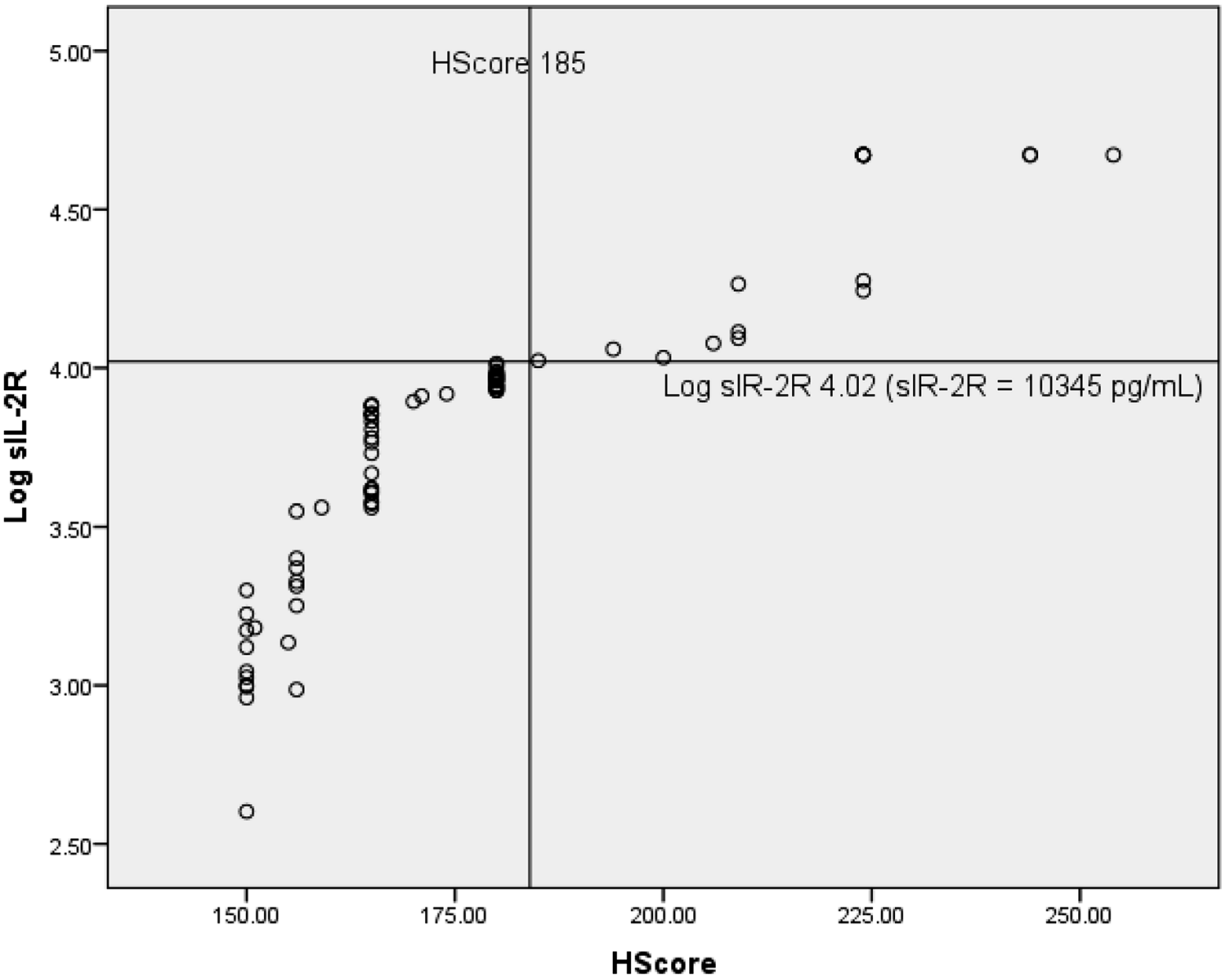
Scatter plot of Log sIL-2R and HScore with cut offs shown at Log sIL-2R of 4.02 (10,345 pg/mL) and 185 for HScore

Eighty patients admitted with dengue were analyzed. Fifty five (68.8%) of them were males and 25 (31.2%) were females. Patients did not receive Antibiotics or steroids at the time of sampling for sIL-2R. Some have received antibiotics and/or steroids subsequently. Median, IQ range and Min–Max range of Age, Maximum Temperature, Lowest Hemoglobin, Lowest WBC count, Lowest platelet count, Triglyceride, AST, Serum ferritin, HScore and sIL-2R in all these 80 patients are shown in Supplementary Table. Eighteen patients had HLH (HScore more than 185) and 62 patients did not have HLH (HScore less than 185). One patient with dengue and HLH expired. There was no significant difference in age, peak temperature, platelet count, triglyceride, hemoglobin and leucocyte count between HScore > 185 and HScore < 185 groups. Serum ferritin, AST and sIL-2R were significantly higher in those patients with dengue fever and HScore > 185 compared to patients with HScore < 185 (Table 1). sIL-2R correlated very well with HScore (*r* = 0.98, *p* < 0.001) compared to serum ferritin (*r* = 0.51, *p* < 0.001). sIL-2R had an AUROC of 1.00 compared to serum ferritin which had AUROC of 0.694 (95% CI 0.557–0.831) for HScore > 185. A cut-off value of 10,345 pg/mL for sIL-2R had a sensitivity and specificity of 100% for HScore > 185, whereas, a ferritin value of 8613 ng/mL had 67% sensitivity and 55% specificity for HScore > 185. Figure 1 shows the scatter plot of Log sIL-2R Vs HScore and Supplementary Figure shows the scatter plot of ferritin Vs HScore with appropriate cutoffs shown in *x* and *y* axes.

**Table 1:** Clinical and Biochemical parameters among patients with HScore of < 185 and > 185 expressed as Median (IQ range) [Min–Max] for continuous variables and Number (Percentage) for categorical variables

	H Score < 185 (*n* = 62)	H score > 185 (*n* = 18)	*p*
Age (years)	38.5 (28.0–48.25) [16–85]	46.5 (33.75–51.25) [18–70]	0.310
Maximum temperature (Centigrade)	39.8 (39.6–39.9) [39.2–41]	39.75 (39.68–39.83) [39.5–40]	0.686
Hepatomegaly	47 (75.8%)	17 (94.4%)	0.07
Splenomegaly	49 (79.0%)	15 (83.3%)	0.49
Lowest WBC count (mm^3^)	3800 (2900–4655) [800–10100]	3250 (2475–4525)[1000–6900]	0.113
Lowest Hb(gm/dL)	14.2 (13.1–15.0) [10–18.2]	13.15 (11.9–15.0) [8.4–16.3]	0.201
Lowest platelet count (mm^3^)	17,000 (11,750–31,500) [4000–13200]	16,000 (11,500–20,750) [9000–55000]	0.467
Triglyceride (mg/dL)	184 (87–267.5) [37–301]	202 (155–268) [121–401]	0.322
AST (U/L)	175.5 (94.7–314.3) [47–3975]	332 (171.3–742.3) [99–1470]	0.011
S. ferritin (ng/mL)	7583.5 (4144.3–10794.3) [1192–42650]	11230.5 (7312.8–18475.5) [3857–65177]	0.013
sIR-2R (pg/mL)	6210 (2107.5–8743.8) [400–10330]	32917.5 (12292.5–46990.0) [10540 ≥ 46990]	< 0.001

There was no statistically significant correlation between sIL-2R and Hematocrit (*r* = – 0.05, *p* = 0.657) or sIL-2R and platelet count (*r* = – 0.054, *p* = 0.635). Values of sIL-2R were compared among severe and non-severe dengue patients of our study population. Median sIL-2R (IQ range) was 7585 pg/mL (3587.5–9597.5) among non-severe dengue patients compared to Median (IQ range) of 9280 pg/mL (8535.0–46,990) in patients with severe dengue. Higher level of sIL-2R in severe dengue patients was statistically significant (*p* = 0.029) by Mann–Whitney *U* test. High sIL-2R showed specificity of 81.2% (56/69) and Negative predictive value of 90.3% (56/62) for severe dengue. There was no statistically significant difference in sIL-2R levels between males and females. (8700 (4060–10,330) pg/mL among males compared to 5380 (1837–9635) pg/mL among females, *p* = 0.084).

## Discussion

In this cross-sectional study of patients with dengue fever, we have shown that sIL-2R level above 10,345 pg/ml appears to be a single biomarker which can identify development of HLH, with very high degree of sensitivity and specificity. sIL-2R appears to be a superior biomarker to ferritin, for diagnosing dengue associated HLH. Previous studies have reported conflicting conclusions regarding the utility of sIL-2R in adult HLH. Hayden et al. reported that sIL-2R was a very useful test for Adult HLH [10], whereas subsequent studies did not confirm this observation [12]. Non-HLH patients in Hayden’s study had a mean HScore of 87.5 resulting in very low pretest probability of HLH and this might have overestimated the diagnostic accuracy of sIL-2R in this group of patients [12]. In our study, mean HScore of patients without HLH was 166, which is close to the cut-off of 185 and still the results were impressive. Inclusion of single disease entity (i.e. Dengue fever) which results in homogeneous etiology of HLH, might be the explanation for this observation. Inflammatory responses are qualitatively and quantitatively different in various etiologies and hence the thresholds of the inflammatory markers for defining HLH could be different in heterogeneous etiologies of HLH. It has been noted that sIL-2R levels are higher in malignancy associated HLH compared to infection induced HLH (20,241 Vs 9720 U/mL) [10]. Disease specific cutoffs might improve the diagnostic accuracy of sIL-2R in infection-associated HLH. Naymagoen et al. observed that sIL-2R is a failed test for HLH among adults based on their study of patients consisting of heterogeneous disease entities [12], but if done in homogeneous population of dengue patients, it could be an extremely successful single test.

Serum ferritin has been an indicator of a higher inflammatory state in many studies. Traditionally, febrile patients with cytopenia, organomegaly and extremely elevated serum ferritin were the patients where HLH was initially suspected [9], but ferritin lacks specificity in infection-associated HLH [16, 17]. Specificity of S. ferritin for diagnosing HLH was only 55% in our study. Ferritin is released from activated macrophages which would be an indicator of fully evolved cytokine storm whereas elevated sIL-2R indicates the initial stages of activation of the T lymphocytes. Rise of sIL-2R has often been noted to precede the development of clinical and hematological parameters [13].

Conventional criteria use multiple inputs to diagnose HLH. Fibrinogen is not estimated in most of the labs in resource limited settings. Accurate measurement of triglyceride requires the sample to be collected after overnight fasting. NK cell activity is measured in research labs only. Bone marrow changes of HLH are not seen in early stages of the disease and they are neither sensitive nor specific for HLH. Moreover, 91% of dengue HLH patients have coagulopathy [3], which might preclude performance of bone marrow examination. sIL-2R estimation can be done by ELISA method which is available in most of the laboratories and input costs are higher by INR 50 only, per test compared to the input cost of estimating ferritin. sIL-2R can be a superior replacement for ferritin as a single biomarker for the early identification of HLH in dengue fever.

Ideal cut-off of sIL-2R has been under a lot of discussion. Initial Histiocyte Society 2004 criteria had a cut-off of 2500 U/mL. Subsequently many researchers have observed that there is a wide variation in the values of sIL-2R among kits from different manufacturers [4, 13]. Kit specific mean values should be considered to determine the ideal cut-off of sIL-2R [13]. Even for parameters like triglyceride and fibrinogens, the original Histiocyte Society 1991 criteria did mention cut-off of mean + 3 standard deviation [18]. The mean value for sIL-2R for DIACLONE kit used in the study is 4051(SD 1998) pg/mL. Mean + 3 standard deviation would be (99 th percentile of the normal individuals) 4051 + 3 × 1998 = 10,045 pg/ml which is very close to the value we got (10,345 pg/mL) by analysing the coordinates of ROC curve. Similar cut-off values were reported by Guleria et al. for identifying HLH is systemic juvenile idiopathic arthritis. sIL-2R cut-off level of 10,385 pg/mL was found to have sensitivity of 100% and specificity of 96.7% in differentiating Macrophage Activation Syndrome from disease flare [19]. The cut-off derived by our study (10,345 pg/mL) is very close to the values observed by Guleria et al. The cut-off for sIL-2R identified in this study should be seen as a preliminary finding only. Larger studies with complete parameters of HScore in the dataset could yield a definitive cut-off for sIL-2R.

Results of this study have immense clinical implications. With a single test, the possibility of a dengue fever patient progressing to HLH can be quickly and easily identified and prognosticated. Early treatment is very crucial in the management of HLH; a high degree of suspicion should prompt the initiation of treatment. In acute infection-associated HLH, early markers like sIL-2R are likely to be more relevant than ferritin which reflects the damage which has already developed. Estimation of a single biomarker like sIL-2R would help in deciding early empirical treatment for HLH.

Not having all the parameters in the calculation of HScore is a limitation of the study. Bone marrow and fibrinogen levels were not available for the calculation of the score. Fibrinogen and Marrow hemophagocytosis contribute a total of 65 points (30 + 35) out of a total possible score of 337 in HScore which is about 19% of the total score. However, those patients who have reached a score of 185 with the available parameters will definitely have score > 185 even after adding bone marrow and fibrinogen levels. There is a small possibility that some of those with less than the cut-off score might become positive with the addition of these parameters. Even if some of the subjects with less than a score of 185 turn out to be positive after adding bone marrow findings and fibrinogen levels, it would reduce the negative predictive value, but the positive predictive value will remain the same. Since sIL-2R had 100% positive predictive value, those who turn positive with sIL-2R can immediately be started on appropriate treatment without waiting for detailed and complex evaluation.

Future studies could involve large numbers of dengue patients in a prospective manner, to evaluate the practicality of using sIL-2R in routine practice. Similar approach could be extended to other viral infections too, including diseases like COVID-19, where development of HLH has been proposed as one of the mechanisms for clinical deterioration. Efficacy of sIL-2R based empirical management of HLH in dengue fever can only be confirmed by large, multi-centric, randomised controlled trials.

## Conclusion

sIL-2R could be a single most useful biomarker to differentiate dengue fever patients who are likely to progress to HLH, from those that are not. In acute infection-associated HLH, an early marker like sIL-2R is likely to be more relevant than serum ferritin, which peaks much later in the inflammatory process, since early treatment is vital to prevent organ damage.

## Supplementary Information

Below is the link to the electronic supplementary material.

Supplementary file1 (DOCX 60 kb)
