# Supplementary material for: Serum soluble interleukin-2 receptor (sIL-2R) is an accurate biomarker for dengue-associated hemophagocytic lymphohistiocytosis syndrome diagnosed by Hscore
Source: Infection. 2022 Aug 23;51(2):433–8. doi: 10.1007/s15010-022-01906-8 (PMC9398040; doi:10.1007/s15010-022-01906-8)
Supplement: Supplementary file 1 — Supplementary file1 (DOCX 60 kb) [file 15010_2022_1906_MOESM1_ESM.docx]

Supplementary Table

Clinical and Biochemical variables of all Dengue patients (80): Median, IQ range, Min-Max for continuous variables and Number(Percentage) for categorical variables

|  | Median (IQ range) | IQ Range | Min-Max |
| --- | --- | --- | --- |
| Age (yrs) | 41 | 28.25-49.0 | 16-85 |
| Maximun Temperature (⁰C) | 39.8 | 39.6 – 39.9 | 39.2-41 |
| Hepatomegaly | 64(80%) |  |  |
| Splenomegaly | 64(80%) |  |  |
| Lowest WBC count (mm^3^) | 3800 | 2600 - 4600 | 800-10100 |
| Lowest Hb (gm/dL) | 14.1 | 12.8-14.9 | 8.4-18.2 |
| Lowest Platelet Count (mm^3^) | 16500 | 12000 - 27250 | 4000-132000 |
| S. Ferritin (ng/mL) | 8613 | 5125-12719.5 | 1192-65177 |
| Triglyceride (mg/dL) | 199 | 146-260 | 37-401 |
| AST (U/L) | 189.5 | 102.3-339.0 | 47-3975 |
| sIR-2R (pg/mL) | 7995 | 3635-10050 | 400 - ≥46990 |
| HScore | 170.5 | 160.5-180 | 150-254 |

Supplementary Figure

Supplementary Figure: Scatter plot of ferritin and HScore with cut offs shown at 8613 ng/mL for ferritin and 185 for HScore
